# Pangenome characterization and analysis of the *NAC* gene family reveals genes for *Sclerotinia sclerotiorum* resistance in sunflower (*Helianthus* *annuus*)

**DOI:** 10.1186/s12863-024-01227-9

**Published:** 2024-05-01

**Authors:** Yan Lu, Dongqi Liu, Xiangjiu Kong, Yang Song, Lan Jing

**Affiliations:** College of Horticulture and Plant Protection, Inner Mongolia Agricultural University, Hohhot, China

**Keywords:** Sunflower, *NAC* gene family, Pangenome characterization, Sclerotinia head rot resistance, Basal stalk rot resistance

## Abstract

**Background:**

Sunflower (*Helianthus* *annuus*) is one of the most important economic crops in oilseed production worldwide. The different cultivars exhibit variability in their resistance genes. The NAC transcription factor (TF) family plays diverse roles in plant development and stress responses. With the completion of the *H. annuus* genome sequence, the entire complement of genes coding for NACs has been identified. However, the reference genome of a single individual cannot cover all the genetic information of the species.

**Results:**

Considering only a single reference genome to study gene families will miss many meaningful genes. A pangenome-wide survey and characterization of the NAC genes in sunflower species were conducted. In total, 139 *HaNAC* genes are identified, of which 114 are core and 25 are variable. Phylogenetic analysis of sunflower NAC proteins categorizes these proteins into 16 subgroups. 138 HaNACs are randomly distributed on 17 chromosomes. SNP-based haplotype analysis shows haplotype diversity of the *HaNAC* genes in wild accessions is richer than in landraces and modern cultivars. Ten *HaNAC* genes in the basal stalk rot (BSR) resistance quantitative trait loci (QTL) are found. A total of 26 *HaNAC* genes are differentially expressed in response to Sclerotinia head rot (SHR). A total of 137 *HaNAC* genes are annotated in Gene Ontology (GO) and are classified into 24 functional groups. GO functional enrichment analysis reveals that *HaNAC* genes are involved in various functions of the biological process.

**Conclusions:**

We identified *NAC* genes in *H. annuus* (HaNAC) on a pangenome-wide scale and analyzed *S. sclerotiorum* resistance-related NACs. This study provided a theoretical basis for further genomic improvement targeting resistance-related *NAC* genes in sunflowers.

**Supplementary Information:**

The online version contains supplementary material available at 10.1186/s12863-024-01227-9.

## Background

Many important cellular processes in plants are controlled by transcriptional regulation, such as signaling transduction, cellular morphogenesis, and various stress responses [1]. Regulation of gene expression requires a group of proteins known as transcription factors (TFs).

Transcription factors, which belong to a highly diverse family of proteins, generally function in protein complexes composed of multiple subunits. The *NAC* (NAM, ATAF_1/2_, and CUC_2_) gene family encodes one of the largest and most important TFs in plants [2]. It has been reported that numerous NAC TFs take part in the regulation of a series of biological processes related to plant growth and development, including embryo and root development [3, 4], cell division [5], flowering [6], cell wall synthesis [7], leaf senescence [8], and response to abiotic and biotic stress [6, 9]. Because of their significance in plant complex life activities, genome-wide screening of NAC was performed in many plants like tobacco [10], poplar [11], cotton [12], foxtail millet [13], Arabidopsis, and rice [14].

As a large number of reference genomes have been released, genomic approaches can be employed to identify specific genes and study the correlations between candidate genes and heritable traits [15]. However, a single reference genome is unable to cover the full genetic information of a species due to structural variations which comprise deletions, insertions, translocations, inversions and duplications. These variations are often related to important agronomic traits [16, 17]. The reference genome of plant species is often derived from cultivated species, which cannot represent the rich genetic diversity of wild species, limiting the study of crop evolutionary and domestication history at higher breadth and depth. Therefore, conducting pangenomics analysis becomes crucial to ensure a comprehensive representation of genomic diversity within a species. Pangenomes have been created for many plant species, such as soybean [18, 19], maize [20], Brassica rapa [21], rice [22], Brassica oleracea [23], bread wheat [24], sunflower [25], and Brassica napus [26, 27].

The concept of a "pangenome" encompasses the entirety of genes in a species, without redundancy. It comprises two categories: core genes and variable genes. Core genes are found in all or nearly all individuals, while, variable genes occur only in certain individuals [16]. Variable genes include two variable types: copy number variations (CNVs) and gene presence/absence variations (PAVs) [28, 29].

Sunflower (Helianthus annuus L.) is an important source of edible oil and the seeds are used for food as well. It is produced worldwide because of its ability to grow and adapt in the most rigid environments. With the completion of the *H. annuus* genome sequence [30], the entire complement of genes coding for NACs has been identified and described [31]. The traditional reference genome (v1.0) provides a foundation for discovering these *NAC* genes. However, due to the effects of environmental factors, different individuals have formed extremely special genetic traits, and the reference genome of a single individual cannot represent the genetic diversity of the species in the process of evolution, leading to a loss of many meaningful genes. 

In this study, *NAC* genes in *H. annuus* (HaNAC) on a pangenome-wide scale were identified, PAVs were detected, and the phylogenetic characteristics and distribution on chromosomes were analyzed. We studied single nucleotide polymorphisms (SNPs) and haplotype variation of *NAC* genes to understand the genetic diversity among different populations. In order to better understand the features of disease resistance-related NACs, the *NAC* genes in QTL regionfor Sclerotinia basal stalk rot (BSR) resistance were surveyed. Furthermore, we analyzed the expression of the *NAC* gene in response to Sclerotinia head rot (SHR). This study may provide clues in identifying disease resistance-related genes in this important crop.

## Materials and methods

### Pangenome

The *H. annuus* pangenome was described by Hübner et al. [25]. It was generated by sequencing 493 accessions, including 287 cultivated lines, 17 Native American landraces, and 189 wild accessions representing 11 compatible wild species. 

### Retrieval of *NAC* genes

Sunflower protein sequence data were obtained from the sunflower genome database (https://sunflowergenome.org/pangenome-data/). The Hidden Markov Model (HMM) profile of the NAC domain (PF02365) was downloaded from the Pfam database (http://pfam.xfam.org) and used to identify potential NAC genes from the sunflower genome via HMMER 3.3.2 software (http://www.hmmer.org/), with an E-value threshold of 10^–5^. Only those proteins predicted to contain the NAM domain by the online tool SMART (http://smart.embl-heidelberg.de/smart/batch.pl) were considered as candidate NAC members. *Arabidopsis* NAC protein sequences were downloaded from the *Arabidopsis* genome, TAIR 10.0 release (http://www.arabidopsis.org/). Gene PAV was discovered across the cultivated gene pool using the SGSGeneLoss package [32]. 

### Phylogenetic tree construction

The NAC protein sequences of sunflower and *Arabidopsis* were aligned using the program MAFFT 7.490 [33] with default parameters, and the tree construction was carried out by the Maximum Likelihood method using the program FastTree 2.1.11 (http://www.microbesonline.org/fasttree/). NAC family genes were classified into several subgroups based on similarities in NAC domain structures [34].

### Density/distribution of the *NAC* gene on sunflower chromosomes

The NAC density/distribution was plotted using the rtracklayer package, karyoploteR package, and RColorBrewer package in Rscript (v4.0.3).

### Gene–CDS–haplotype (gcHap) analysis of the *NAC* gene family

Vcftools 0.1.15 was used to screen the VCF files of Sunflower to obtain SNPs by further removing rare alleles with a missing rate of > 0.4 [35]. gatk_vcf_to_haplotype.pl (https://github.com/zhuochenbioinfo/VCF2HAP) was used to identify gcHapin all samples, and then to calculate the number of haplotypes in different populations.

To assess gene diversity across different populations, Shannon's equitability (*E*_*H*_) [36] was calculated using the gcHap (gene–coding sequence–haplotype) data. The formula was:

$$E_H=\frac1{\text{ln}N}(-\sum pi\ln pi)$$

where *p*_*i*_ is the proportion of the *i*th gcHap of a gene, *N* is the population size, and ln*N* is the maximum possible diversity of a gene. *E*_*H*_ value ranges between 0 and 1.

Nei’s genetic identity (*I*_*Nei*_) [37] was used to measure the genetic differentiation among populations. For each gene, *I*_*Nei*_ between two populations was estimated with the gcHap data. The formula was:

$${I}_{Nei}= \frac{\sum { X}_{i} {Y}_{i}}{\sqrt{\sum { X}_{i}^{2}{ Y}_{i}^{2}}}$$

*Xi* and *Yi* represent the frequencies of the *i*th gcHap of a gene in populations X and Y, respectively.

*E*_*H*_ and *I*_*Nei*_ were visualized using the ggplot2 package and ggpubr package in R 4.0.3 [38].

### Identification of NAC-genes conferring Sclerotinia BSR resistance

A total of six QTLs responsible for quantitative resistance to BSR have been identified in a sunflower recombinant inbred line population, one of each on linkage groups (LGs) 4, 9, 10, 11, 16, and 17 [39]. BLAST was used to compare the gene sequences at both ends of these QTLs with reference genome sequences (HA412-HO.v1.1). According to *HaNAC* and QTL positions in the reference genome, possible candidate *HaNAC* genes related to BSR are explored.

SNP data for the *H. annuus* genome and pangenome extra contigs were downloaded from (https://sunflowergenome.org/pangenome-data/HelianthusVariants.vcf.gz).

SNPs flanking the known Sclerotinia BSR resistance QTL regions were collected from the literature [39]. Waterfall plots were drawn using Variant Effect Predictor 88.13 [40], GenVisR 1.11.3 [41], vcftools 0.1.15 [42] and R 4.0.3.

### Analysis of RNA-seq data of SHR

SHR is caused by the necrotrophic fungus *Sclerotinia sclerotiorum*. Fass et al. [43] studied gene expression at the early stages of infection (0, 4, and 8 dpi) in one susceptible (H89) and two tolerant inbred lines (HA853, RK416) inoculated with the pathogen in field conditions.

RNA-seq data were downloaded from NCBI and SRA accession number was SRP219154 [43]. The fastq-dump tool in the SRA Toolkit 2.10.0 (http://www.ncbi.nlm.nih.gov/Traces/sra/sra.cgi?view=toolkit_doc&f=fastq-dump) was used to Convert SRA files to fastq files. Fastp 0.20.1 was used to trim low-quality bases (average Q-score below 20) and adaptor sequences in raw data [44]. The RNA-seq clean data of each sample were mapped to the sunflower pan-genome using HiSAT2 2.1.0 [45]. FPKM (Fragments Per Kilobase of exon model per Million mapped reads) value of *NAC* genes was calculated. DESeq2 1.32.0 [46] was used for differential expression analysis, |log2 fold change |≥ 1 and p < 0.05 were set as the threshold to determine differentially expressed genes. A heatmap of Log2 (FPKM + 1) values was generated using the ComplexHeatmap package (2.6.2, https://bioconductor.org/packages/release/bioc/html/ComplexHeatmap.html) in R 4.0.3. 

We analyzed the differential expression of *NAC* genes in each inbred line (IL)-time point combination. A total of 27 combinations was shown in Table S1.

### Gene ontology (GO) annotation and enrichment analysis

All HaNAC sequences were compared against the sequences in the UniProt database using the BLASTP with an E-value cutoff of 1e-5. The Retrieve/ID Mapping tool was used (https://www.uniprot.org/uploadlists/) to convert UniProt IDs to GO IDs for HaNAC GO annotation. The agriGO was used for GO enrichment analysis (http://bioinfo.cau.edu.cn/agriGO/analysis.php). All genes in the sunflower pan-genome were used as background. GO enrichment results were visualized using Cytoscape 3.8.0 [47].

## Results

### Pangenome-wide identification of NAC family genes

A total of 139 NAC-encoding genes are identified in the *H. annuus* pangenome, including one *NAC* gene (*HaNAC139*) that is not present in the reference genome assembly. Of the 139 NAC genes, 114 (82.01%) are core genes (found across > 95% of the accessions) and 25 (17.99%) are dispensable genes of which 20 were found in > 5% ~  < 95% of the accessions, and 5 were rare genes that were found in < 5% of the accessions. The detailed information on the 139 *NAC* gene sequences is shown in Table S2.

### Phylogenetic analysis of NAC gene family in sunflower and *Arabidopsis*

**Fig. 1:**
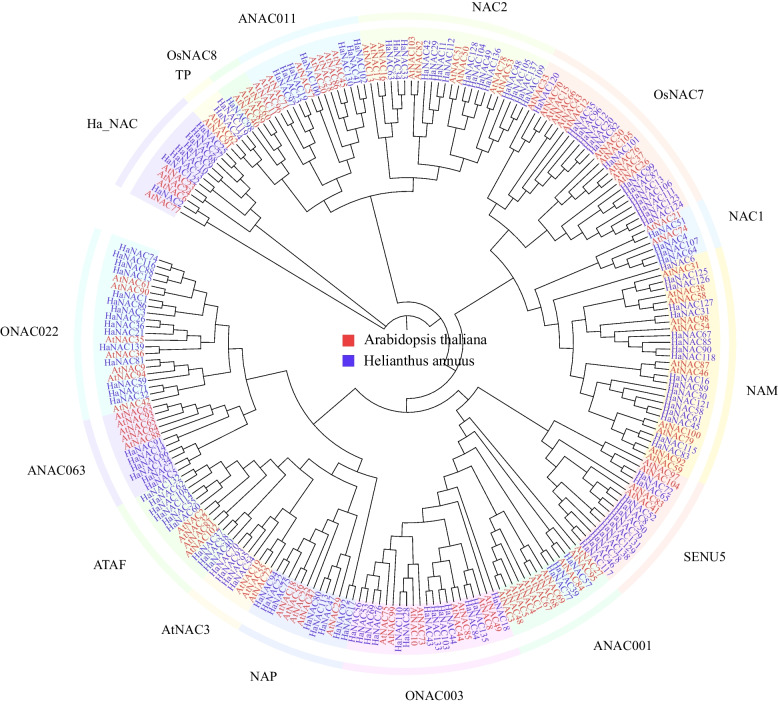
A phylogenetic tree of sunflower and *Arabidopsis* NAC proteins. The amino acid sequences of NAC proteins were aligned using MAFFT 7.490, and a phylogenetic tree was generated using the Maximum Likelihood method of FastTree 2.1.11. NAC family genes were classified into different subgroups based on similarities in NAC domain structures [34]. Blue and red fonts denoted sunflower and *Arabidopsis* NACs, respectively. All NACs were classified into 16 subgroups (different colors for each clade)

To investigate the phylogenetic relationship among the HaNAC family members, a phylogenetic tree is constructed based on the alignment of 240 full-length protein sequences from sunflower and *Arabidopsis.* As indicated in Fig. 1, the 139 HaNACs are divided into 16 subgroups. Since AtNAC6, 23, 24, and 77 don’t belong to any group, *HaNAC* genes highly similar to these genes are assigned to the Ha_NAC subgroup. The largest clade is the NAM subgroup containing 18 HaNACs, while the OsNAC8 subgroup constitutes the smallest clade with only one HaNAC98.

**Fig. 2:**
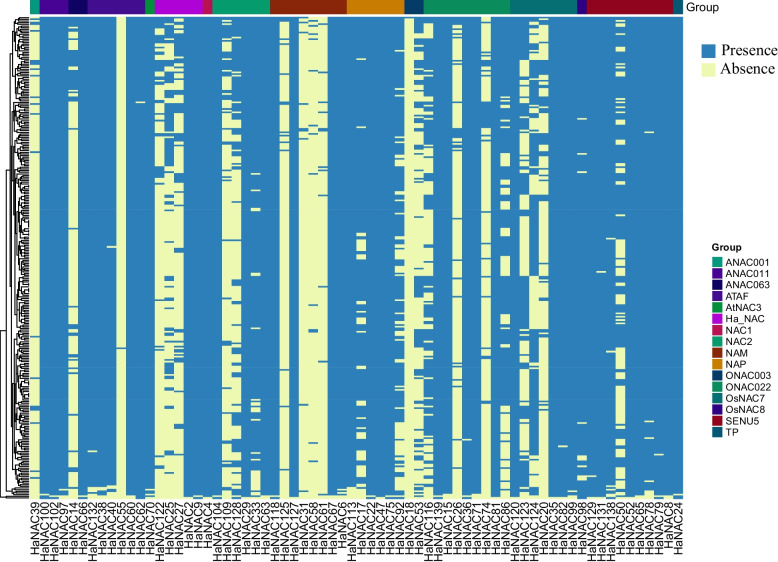
Heat maps of gene presence/absence variation (PAV) in 290 sunflower lines

All subgroups of the HaNAC family contain variable genes (present only in some individuals) (Fig. 2). In total, the absence of 68 genes occurs in 5074 accessions (Table S3). In a total of 290 accessions, *HaNAC18* and *HaNAC55* are absent in 289 accessions, *HaNAC31* is absent in 281 accessions, and *HaNAC61* is absent in 278 accessions.

The absence of variable genes in subgroups ANAC011, AtNAC3, NAC1, OsNAC8, and TP occur in few accessions, with 5, 3, 1, 11, and 1 respectively, indicating that these five subgroups are relatively stable among all groups. On the contrary, the gene absence in subgroups ANAC001 and ONAC003 occurs in a higher proportion of accessions, with 269 (~ 92.8%) and 484 (~ 83.4%), respectively, suggesting that these two groups are the most unstable among all groups and might endure strong selection pressure during sunflower domestication and breeding.

### HaNAC gene distribution on sunflower chromosomes

**Fig. 3:**
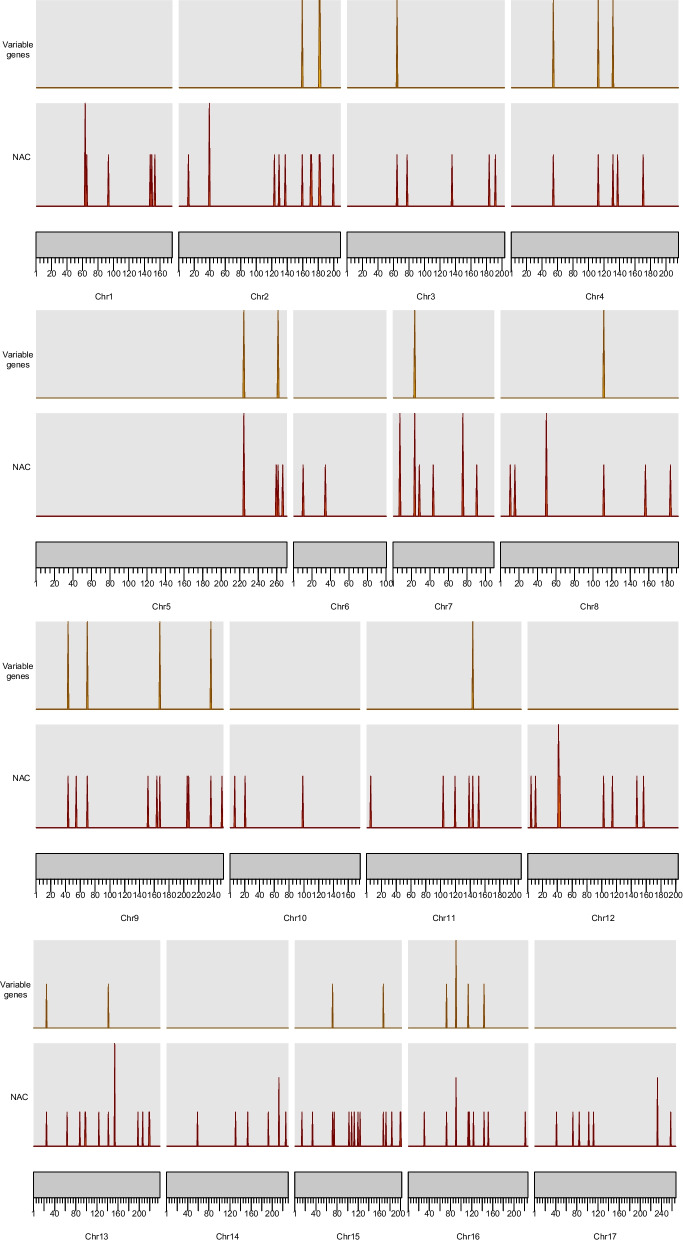
The distribution of *NAC* genes vs variable *NAC* genes on sunflower chromosomes. These densities were normalized by the genome-wide maximum of each measurement so that they peak at 1. The ruler at the bottom was used to show the size of each chromosome

We mapped the 138 *HaNAC* genes on all 17 chromosomes (Chr 1 to Chr17) and named them from *HaNAC1*-*HaNAC138* according to their chromosomal locations. As shown in Fig. 3, HaNAC sequences distribute unevenly over all chromosomes. Chr13 and Chr15 have a maximum of 14 *HaNACs* (~ 10.1%), respectively, whereas only 2 *HaNACs* (~ 1.4%) are located on Chr6. Chr5 has the longest size of 271 Mb, but *NAC* genes are distributed only in the region between 220 and 271 Mb. Of the 138 *HaNACs*, 25 variable genes (dispensable genes and rare genes) are located on 11 chromosomes. No variable genes are located on Chr1, Ch6, Chr10, Chr12, Chr 14, and Chr 17, indicating that the *NAC* genes on these chromosomes are relatively stable during evolution and domestication. Half or more than half of the *NAC* genes on Chr4 and Chr16 are variable genes, suggesting that the *NAC* genes on these two chromosomes probably have undergone selection during sunflower domestication and diversification.

### Analysis of gene–CDS–haplotypes in sunflower *NAC* gene family

We investigated the SNPs and haplotype constructed with adjacent SNPs in *HaNAC* genes. According to SNP information provided by Hübner et al. [25], 3247 SNPs are discovered within the CDS region of 108 *HaNACs*. No SNPs are found within the remaining 31 *HaNAC* genes (Table S4), which may be involved in some basic biological processes and serve as housekeeping genes.

**Fig. 4:**
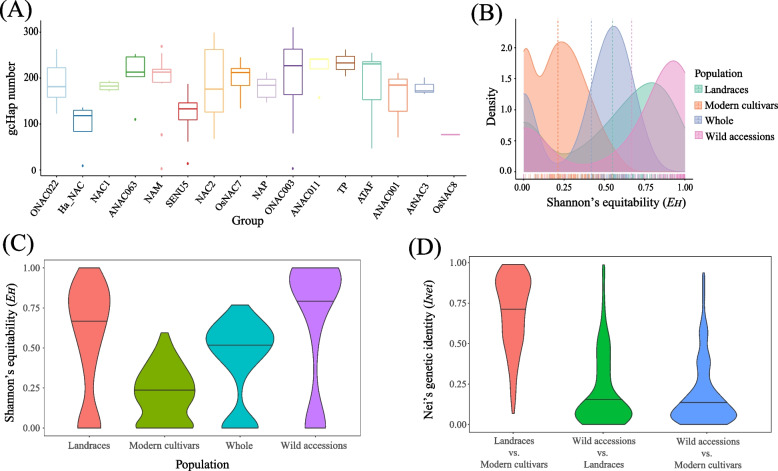
gcHap numbers, Shannon’s equitability (*E*_*H*_) and Nei’s genetic identity (*I*_*Nei*_) of sunflower NAC family among different populations. **A** Distribution of gcHap number (gcHapN) of all 139 *NAC* genes in different phylogenetic groups. **B** Frequency distribution of *E*_*H*_ in landraces, wild accessions, modern cultivars and whole populations. **C** *E*_*H*_ distribution in four different populations. **D** *I*_*Nei*_ distribution of landraces vs modern cultivars, wild accessions vs modern cultivars, and wild accessions vs landraces

We made statistical analysis on the haplotypes in genes of different phylogenetic groups (Fig. 4A), and our results show that haplotypes are the most abundant in group ANAC011, while the least in group OsNAC8. The *NAC* gene in ANAC011 has the largest haplotype diversity, suggesting that these genes play different regulatory roles in different sunflower accessions. However, the *NAC* gene in OsNAC8 showed the least haplotype variation among different accessions, which may be due to its conserved function.

We calculated *E*_*H*_ to evaluate genetic diversity within populations (Fig. 4B, C). In general, genes with lower *I*_*Nei*_ values tend to make a greater contribution to population differentiation, while genes with higher *I*_*Nei*_ values have less impact. Furthermore, when the *E*_*H*_ value of a gene is lower, it suggests lower genetic diversity among individuals. Conversely, a higher *E*_*H*_ value indicates greater genetic diversity [35]. The *E*_*H*_ density distribution of HaNAC in wild accessions is closer to the right (larger *E*_*H*_ value) than that of other populations (Fig. 4B), indicating that haplotype diversity of the *HaNAC* gene in the wild accessions is richer. The peak value of *E*_*H*_ density in modern cultivars is closer to the left (lower *E*_*H*_ value), indicating that, the genetic diversity of the *HaNAC* gene decreases after a long-term domestication. Figure 4C shows the *E*_*H*_ value of each population, and the mean value of the wild accessions is the largest, followed by landraces, whole and modern cultivars decreasing in their genetic diversity.

To compare the genetic diversity between different populations, we calculated *I*_*Nei*_ (Fig. 4D). Wild accessions vs landraces and wild accessions vs modern cultivars both have lower *I*_*Nei*_ values, indicating that the SNP-haplotypes of the *HaNAC* gene in the wild population are significantly different from those in the other two populations. However, the *I*_*Nei*_ values of landraces vs modern cultivars are mostly above 0.5, indicating that there is little haplotype difference between the *HaNAC* genes in these two populations.

### Introgression in sunflower *NAC* gene family

According to the sunflower pangenome data [25], we verified whether the *NAC* gene family has introgression. The results show that HaNAC26 in cultivated sunflower association mapping (SAM) population has gene introgression from *H. argophyllus* and *H. neglectus*, and gene introgression from these two neighboring species into *H. annuus* arises in two samples.

### *NAC* genes in Sclerotinia BSR resistance QTL regions

**Fig. 5:**
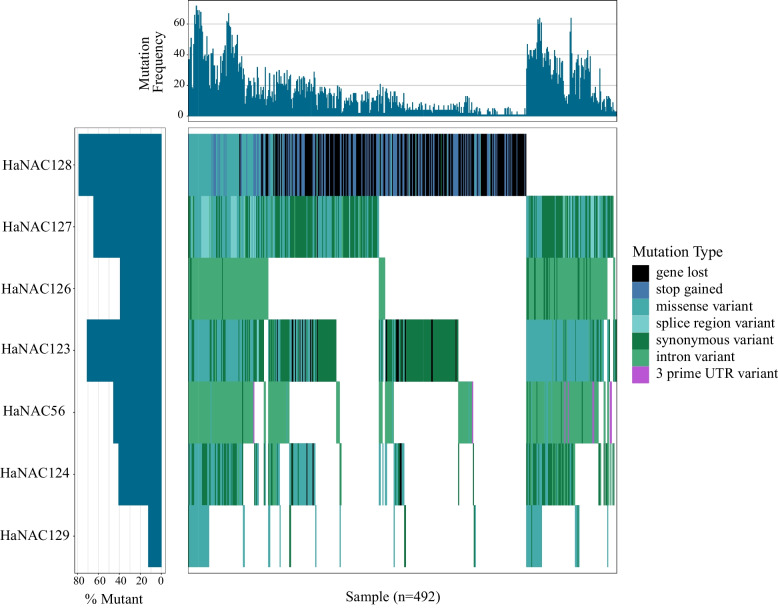
Waterfall plot of *HaNAC* variation in the QTL region in 492 sunflower accessions

Ten *HaNAC* candidates are identified at loci Qbsr-4.1, Qbsr-9.1, and Qbsr-16.1 (Table 1). *HaNAC26* locates at loci Qbsr-4.1, *HaNAC56* at loci Qbsr-9.1 and *HaNAC122*, *HaNAC123*, *HaNAC124*, *HaNAC125*, *HaNAC126*, *HaNAC127*, *HaNAC128*, *HaNAC129* at loci Qbsr-16.1. Of them, *HaNAC56*, *HaNAC126*, and *HaNAC127* are core genes, and the rest are dispensable ones. Combining with SNP and PAV information in the sunflower pangenome, the variation of 10 *HaNAC* genes in 492 accessions is analyzed (Fig. 5). The sunflower pan-genome contains the genetic information of 493 accessions. Because the *HaNAC* genes in the QTL regions have no SNP markers in accession PPN021, PPN021 is excluded from the analysis.

**Table 1:** The number of *HaNACs* at the Qbsr-4.1, Qbsr-9.1and Qbsr-16.1

Locus	Pseudomolecule	Start (bp)	End (bp)	Length (bp)	HaNAC number
Qbsr-4.1	Chr4	107675011	124808091	17133080	1 (HaNAC26)
Qbsr-9.1	Chr9	135225176	153762638	18537462	1 (HaNAC56)
Qbsr-10.1	Chr10	132441999	136480784	4038785	0
Qbsr-11.1	Chr11	170688742	185809916	15121174	0
Qbsr-16.1	Chr16	45544215	199280159	153735944	8 (HaNAC122、HaNAC123、HaNAC124、HaNAC125、HaNAC126、HaNAC127、HaNAC128、HaNAC129)
Qbsr-17.1	Chr17	150544245	230082427	79538182	0

The genes *HaNAC26*, *HaNAC122*, and *HaNAC125* in the QTL region are not displayed in the waterfall map because they do not contain SNP markers, i.e., they do not have gene variation in 492 accessions, suggesting that these three genes are very conservative. Among the remaining seven *HaNAC* genes, *HaNAC128* shows the largest variant mainly due to the gene loss that is present in 171 samples, indicating that *HaNAC128* is subject to the greatest pressure of PAV selection. Mis-sense variant, synonymous variant, and intron variant are the main variation patterns of the *HaNAC* gene in the QTL region. *HaNAC123* has the highest proportion of synonymous variants, while *HaNAC126* has the highest proportion of intron variants. The *HaNAC129* shows low variation (< 20%), but the majority of variants are missense, indicating that this gene has been strongly and positively selected in some accessions.

### Expression analysis of *HaNAC* gene in response to SHR

Based on the transcriptome data of inoculated (I) and control (N) capitula of three sunflower inbred lines (ILs) [43], we analyzed the differential expression of each IL-time point combination.

**Fig. 6:**
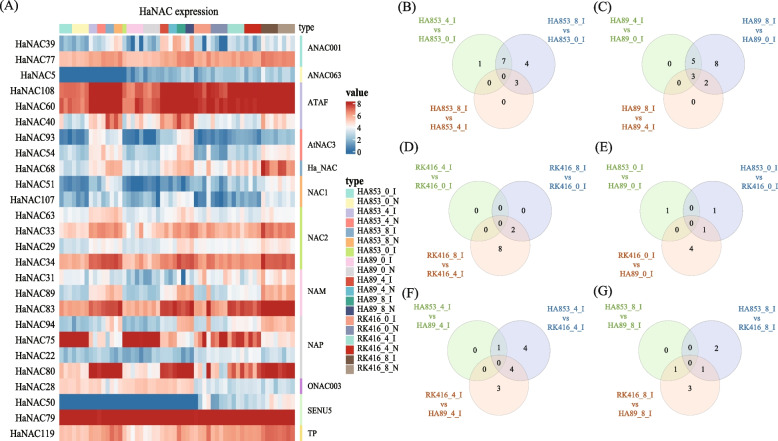
Heatmaps and Venn diagrams. **A** Heatmaps of differentially expressed *HaNAC* genes. **B-G** Differentially expressed *HaNAC* genes in the three datasets through Venn diagrams software (available online: http://bioinformatics.psb.ugent.be/webtools/Venn/). Different colors meant different combinations

A total of 26 *HaNAC* genes were expressed differentially (Fig. 6A, Table S5). *HaNAC* genes in groups ANAC063 and NAC1 showed low expression levels. In addition, among all differentially expressed *HaNAC* genes, the number of *HaNAC*_*S*_ in groups NAP and NAC2 was the largest, indicating that compared with others, the *HaNAC* genes in these two groups might be more involved in response to *S. sclerotiorum*.

All the combinations of I vs N had no differentially expressed gene (DEG) (data not shown). At three time points, HA853 and HA89 had larger numbers of DEGs in 8 dpi vs 0 dpi, 14 and 18 respectively, while 8 DEGs were found respectively in 4 dpi vs 0 dpi (Fig. 6B, C). However, RK416 had 10 DEGs in 8 dpi vs 4dpi, and no DEG in 4 dpi vs 0 dpi (Fig. 6D). The result indicated that HaNACs respond quickly to SHR in HA89 and HA853, while HaNACs respond slowly to SHR in RK416.

There were five DEGs in RK416_0_I vs HA89_0_I (Fig. 6E), seven in RK416_4_I vs HA89_4_I and nine in HA853_4_I vs RK416_4_I (Fig. 6F), five in RK416_8_I vs HA89_8_I and three in HA853_8_I vs RK416-8-I respectively (Fig. 6G), indicating that *HaNAC* genes in different lines responding to *S. sclerotiorum* are different at the same time point.

**Fig. 7:**
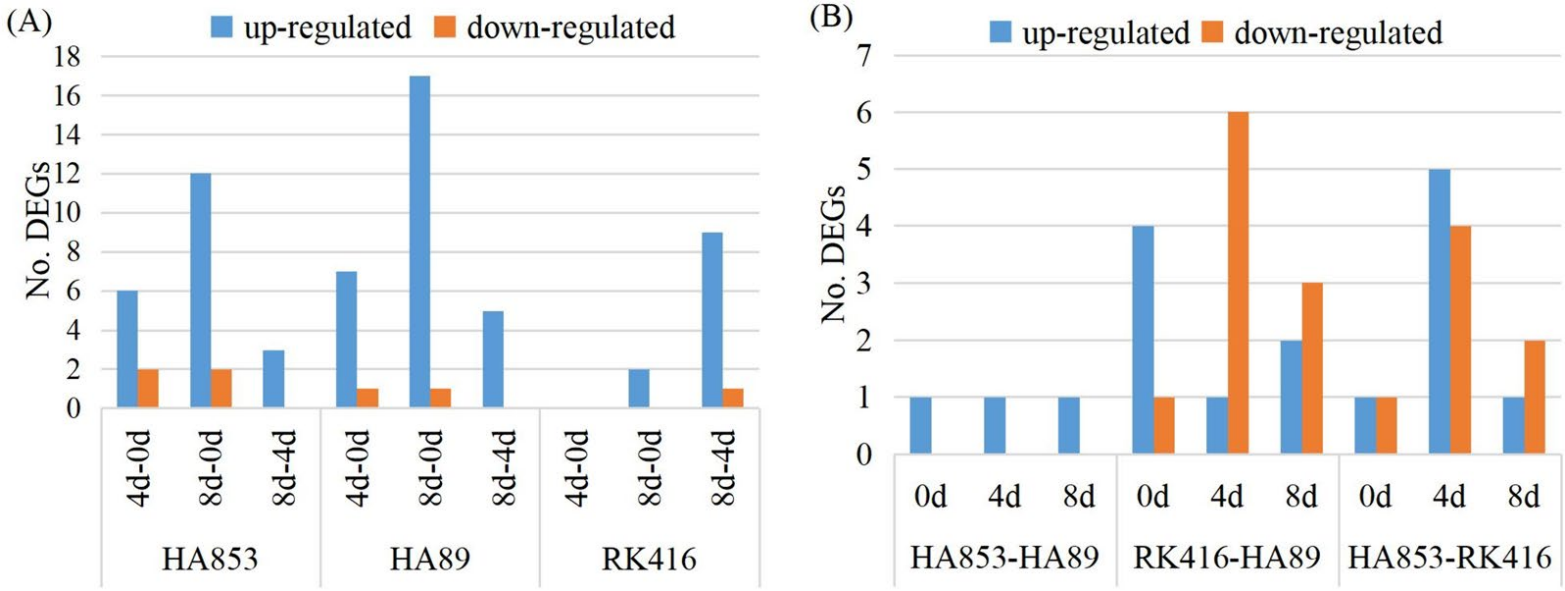
Analysis of DEGs. **A** Number of up- and down-regulated DEGs between IL-time- inoculated samples. **B** Number of up- and down-regulated DEGs between three inoculated lines

Most IL-time point combinations had larger numbers of up-regulated than down-regulated DEGs (Fig. 7A). Figure 7B shows the number of DEGs between three lines.

### Functional annotation and enrichment analysis of *HaNAC* genes

To further understand the function of *NAC* genes in sunflowers, we performed GO annotation and functional enrichment analysis for *HaNAC* genes.

**Fig. 8:**
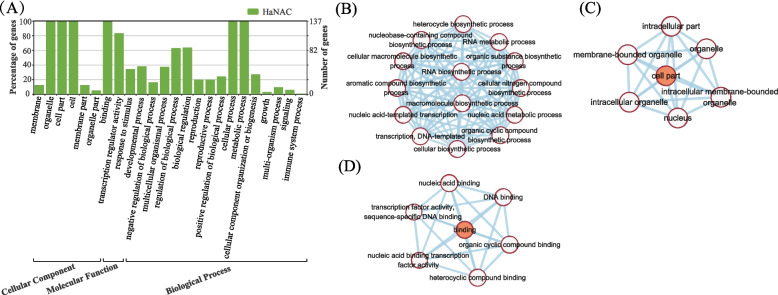
Gene ontology annotation of HaNAC and network diagram of Go terms. **A** Gene ontology annotation of HaNAC. 137 sequences were grouped into three major functional categories and 24 sub-categories. **B** Network diagram of GO terms enriched in biological process. Only the top 10% of GO terms with the lowest FDR (false discovery rate) value were showed. **C** Network diagram of GO terms enriched in cellular component. **D** Network diagram of GO terms enriched in molecular function

A total of 137 HaNACs are annotated in Gene Ontology (GO) and are classified into 24 functional groups, including 15 groups in the biological process, six in cellular component, and three in molecular function (Fig. 8A). Within the biological process, the “metabolic process” (GO: 0008152) and “cellular process” (GO: 0009987) with 137 HaNACs respectively are predominant. In the category of cellular component, the three main groups are “organelle” (GO: 0043226, 137 HaNACs), “cell” (GO: 0005623, 137 HaNACs), and “cell part” (GO: 0044464, 137 HaNACs). The categories “binding” (GO: 0005488) and “transcription regulator activity” (GO: 0140110) are the most common in molecular function, represented by 137 and 114 HaNACs, respectively.

GO functional enrichment analysis reveals that *HaNAC* genes are mainly involved in the biological process (Fig. 8B). Figure 8C shows the network of enriched GO terms in the cellular component category. Figure 8D shows GO terms are enriched in the molecular function category, including DNA binding, transcription factor activity, sequence-specific DNA binding, nucleic acid binding transcription factor activity, nucleic acid binding, organic cyclic and heterocyclic compound binding. These represent the characteristics of the HaNAC proteins as transcription factors, which regulate gene expression via transcription by binding to gene-specific sequences and affect the biological activities of cells.

## Discussion

The NAC family is one of the largest plant-specific TFs that are involved in regulating growth, development, and stress responses. To date, *NAC* genes have been discovered in many plant species represented by 117 genes in *Arabidopsis* [14], 151 in rice [14], 101 in soybean [48], 145 in cotton [12], 104 in tomato [49], 148 in maize [50], 87 in sesame [51], 80 in watermelon [52], 145 in sorghum [53], 85 in sugarcane [54], 91 in cucumber [55], 104 in pepper [56], and 164 in cultivated peanut [57]. Li et al. [31] identified 150 HaNACs in sunflower through genome-wide survey (Ha412HO v1.0). The assembly of the cultivated sunflower pan-genome was guided by the HA412-HO.v1.1 reference sequence. Based on pan-genome data, we identified a total of 139 NACs with intact NAC domains in sunflower. Relatively numerous NACs in the sunflower were speculated to be highly involved in the complex transcriptional regulatory networks of sunflower. Multiple gene duplication events are believed to be responsible for this phenomenon as well.

According to the findings of Hübner et al. [25], the cultivated sunflower pan-genome consists of 61205 genes, with approximately 27% of these genes exhibiting variation across different genotypes. Our analysis found that 82% of 139 *NACs* in the *H. annuus* pangenome are core genes and 18% are dispensable genes (including rare genes). In plants, core genes often play a role in essential metabolic processes, while dispensable genes usually function in stress responses [19, 58–60] which tend to evolve faster under stronger selection [60, 61]. Previous studies have shown polymorphism level is higher in dispensable genes than in core genes [18, 26, 62, 63]. In the *B. oleracea* pangenome, nearly 20% of genes show PAV [23]. In the *Glycine soja* pangenome, dispensable genes account for 20% of the total genesets which exhibit greater variation than the core genome [18]. Sorghum pan-genome also displays large variation in genecontent, with 64% of gene families affected by PAV among genomes [64]. Through pangenomic analysis, we can study the retention and loss of genes during domestication and breeding [65]. In our study, PAV analyses revealed the loss of 5074 *NAC* genes during sunflower domestication and improvement, consistent with the trend found in sunflower domestication [25]. PAV is an important contributor to the studies of genetic diversity, gene identification, and molecular marker development in plants [66]. Understanding the PAV gene could support crop improvement applications and potentially reintroduce the gene into modern varieties [67].

Ooka et al. [34] classified NAC family proteins of *Oryza sativa* and *Arabidopsis thaliana* into two groups and 18 subgroups by sequence similarity. NAC proteins classified in the same groups may have similar functions in events common to monocotyledonous and dicotyledonous plants. Many findings suggest that the NAC proteins in subgroups NAM and NAC1 function in morphogenesis [2, 5], and proteins in the ATAF subgroup share a conserved role in stress responses [68]. NAC proteins in subgroup OsNAC3 (a monocot-specific subgroup) may be involved in monocot-specific responses to stress [34]. Analysis of the alignment of sunflower NAC proteins reveals that the proteins constitute a large family and belong to 16 subgroups (Fig. 1). Because proteins with domains similar in alignment are possible to have similar functions, our results will facilitate further functional analysis of sunflower NAC family genes. Our analysis shows the ATAF group consists of nine *NAC* genes in sunflower which may play a pivotal role in response to stress stimuli.

Sunflower holds global significance as it serves as a crucial oilseed crop, as well as a significant supplier of confectionery seeds and ornamental flowers. The cultivated sunflower are derived from wild *H. annuus* and were domesticated in what is now Central America earlier than 4000 years ago [69]. The wild sunflower is a potential source of cytoplasmic male sterility, and fertility restoration genes have been successfully introduced into cultivated sunflower [70–72]. Hübner et al. [25] reported approximately 10% of the cultivated sunflower pan-genome contains introgression of the wild sunflower-derived gene, and 1.5% of the genes are introduced solely through introgression. Introgressed regions show an overrepresentation of genes associated with biotic resistance. Our analysis finds that introgression also exists in the sunflower *NAC* gene family. *HaNAC26* in the SAM population has gene introgression from two wild annual *Helianthus* species *H. argophyllus* and *H. neglectus*. *H. argophyllus* has been used as a valuable source of disease-resistance genes, which provide resistance against *Puccinia helianthi*, *Plasmopara halstedii*, and *S*. *sclerotiorum* in sunflower [73–75]. Hübner et al. [25] found that introgression is related to the formation of sunflower resistance ability. The introgression phenomenon of *HaNAC26* pointed out in this study reveals the *HaNAC26* is possibly associated with resistance.

The SNPs marker system is extensively used in modern genomics research [76]. Out of 139 *HaNAC* genes, 108 contained SNP markers. SNP-based haplotype analysis indicates that haplotype diversity of the *HaNAC* gene among wild accessions is richer than that in the landraces and modern cultivars implying that selection pressure may lead to the loss of genetic diversity in certain populations during sunflower domestication. Low diversity may have weakened their ability to adapt to the environment. The sunflower gcHap diversity dataset generated in this study would contribute to sunflower basic research and future breeding. Polymorphisms within gene coding regions represent the most important part of the overall genetic diversity. Zhang et al. [35] characterized the gcHap diversity of 45963 rice genes in 3010 rice accessions. They found an average of 226 ± 390 gcHaps per gene in rice populations. Low frequencies of ‘‘favorable’’ gcHaps at most known genes related to rice yield in modern varieties suggest massive potential for improving rice by mining and pyramiding favorable gcHaps. The gcHap data were demonstrated to have greater power for detecting causal genes that affect complex traits. The rice gcHap diversity dataset would facilitate rice improvement in the future.

NAC transcription factors are known to be involved in coordinating responses to attacks by phytopathogens. Overexpression of the eggplant (*Solanum melongena*) transcription factor S*mNAC* suppresses resistance to bacterial wilt pathogen *Ralstonia solanacearum* [77]. Analysis suggests a putative NAC transcription factor Rph7 in barley (*Hordeum vulgare*) mediates the activation and strength of the basal defense response to leaf rust pathogen *Puccinia hordei* [78]. The rice *OsNAC30* mutant lines showed markedly reduced susceptibility to *Xanthomonas oryzae* pv. *oryzae* compared to wild-type plants. Mutation of *OsNAC59* conferred resistance to *Fusarium fujikuroi*, while mutation of *OsNAC101* increased susceptibility to this pathogen [79].

White mold caused by *S. sclerotiorum* is a devastating disease causing servere yield losses in sunflower production. Sunflower white mold has three different types of disease symptoms: BSR, mid-stalk rot (MSR), and head rot. So far, no major gene conferring complete resistance against this pathogen has been identified in cultivated sunflowers.

Crop breeding programs have faced challenges in identifying QTL that provide broad-spectrum resistance, which refers to resistance against various plant pathogens. These QTLs have proven to be elusive targets in breeding efforts. Six QTLs for resistance to BSR have been identified in the sunflower recombinant inbred line (RIL) population [39]. Our analysis data reveals that ten *HaNAC*_*S*_ are located at loci Qbsr-4.1, Qbsr-9.1, and Qbsr-16.1 probably play a regulatory role in BSR resistance. Three *NAC* genes (*HaNAC26*, *HaNAC122*, and *HaNAC125*) in the QTL region contain no SNP markers, therefore they have no variation in 492 accessions, which suggests a stable inheritance trait and valuable targets for breeders.

Identifying NAC candidates within QTL may help future breeding efforts in *H. annuus*. SNP markers tightly linked to resistance are also useful for breeding applications. Identifying both core and variable genes within these regions emphasizes the importance of employing pangenomics in these endeavors.

*S. sclerotiorum* has been reported to infect over 400 plant species [80]. Transcriptomic studies in *B. napus*, *A. thaliana,* and *Glycine max* have shown that defense against *S. sclerotiorum* involves transcription factor families, pathogenesis-related (PR) proteins, cell wall related proteins, as well as genes associated with cellular redox state, and hormone signaling pathways [81–85]. Joshi et al. [83] identified 30 TFs from *B. napus* post-infection with *S. sclerotiorum*, mainly including WRKY, NAC, ethylene response element binding factor (EREBF), MYBs, heat shock factors (HSFs), and C3H zinc finger. The results demonstrated the regulatory roles of plant TFs in response to pathogen challenges.

Fass et al. [43] investigated the transcriptional response of sunflowers to SHR. The analysis of differential gene expression revealed limited overlap among the ILs, indicating genotype-specific regulation of cell defense responses, potentially associated with variations in disease resistance strategies. All three ILs demonstrated an impact on the expression of genes related to cellular redox state and cell wall remodeling, aligning with existing understanding of the initiation of plant immune responses. Based on their data, we find a total of 26 differentially expressed *HaNAC* genes (~ 18.8% of the total *HaNAC* genes) involved in the defense against SHR. Our data analysis demonstrates the existence of diversified transcriptional responses to SHR within sunflower breeding lines and provides new evidence of the significant roles *HaNAC* genes played in response to pathogen challenges.

The GO functional annotation analysis further indicates that ‘binding’ and ‘transcription regulator activity’ are the most common molecular functions of HaNAC transcription factors, while ‘cellular process’ and ‘metabolic process’ are the most common biological processes, which is consistent with the characteristics of transcription factors.

## Conclusion

In this study, we analyzed NACs in an *H. annuus* pangenome using a single reference and whole-genome sequencing data from 492 lines. Our various analyses reveal genomic landscape diversity and discover genes that have been lost during domestication in cultivated sunflowers. Our results highlight the potential of variable genes to be used in genetic structural variation studies for future breeding programs. We identify some novel NACs that may contribute to resistance to Sclerotia white mold. Further genetic manipulation of these resistance-linked QTLs and genes will advance the precision breeding of sunflowers. Overall, the constructed sunflower pan-genome provides an important resource for sunflower improvement and gene discovery. And the findings will aid in furthering our understanding of not only the functions of core, and dispensable genes but also on various topics ranging from a better understanding of the evolutionary dynamics of gene families to genotype–phenotype associations.

### Supplementary Information

**Additional file 1: Table S1.** 27 Sample combinations for differentially expressed gene analysis.

**Additional file 2: Table S2.** List of NAC genes of sunflower retrieved from the sunflower pan-genome database.

**Additional file 3: Table S3.** The absence of 68 genes occurs in 5074 accessions.

**Additional file 4: Table S4.** The number of SNPs and haplotypes in sunflower NAC gene family.

**Additional file 5: Table S5.** 26. Differentially expressed *HaNAC* genes.

## Data Availability

All data generated or analyzed in this study can be found in the supplementary information files and NCBI, https://www.ncbi.nlm.nih.gov/datasets/genome/?taxon=4232.

## Abbreviations

TF: Transcription factor
CNVs: Copy number variations
PAVs: Presence/absence variations
QTL: Quantitative trait loci
SHR: Sclerotinia head rot
GO: Gene Ontology
BSR: Basal stalk rot
HMM: Hidden Markov Model
gcHap: Gene–coding sequence–haplotype
*E*_*H*_: Shannon's equitability
*I*_*Nei*_: Nei’s genetic identity
LGs: Linkage groups
FPKM: Fragments Per Kilobase of exon model per Million mapped reads
IL: Inbred line
SAM: Sunflower association mapping
DEG: Differentially expressed gene
MSR: Mid-stalk rot
RIL: Recombinant inbred line
PR: Pathogenesis-related
EREBF: Ethylene response element binding factor
HSFs: Heat shock factors
