# Supplementary material for: Pangenome characterization and analysis of the NAC gene family reveals genes for Sclerotinia sclerotiorum resistance in sunflower (Helianthus annuus)
Source: BMC Genom Data. 2024 May 1;25:39. doi: 10.1186/s12863-024-01227-9 (PMC11064331; doi:10.1186/s12863-024-01227-9)
Supplement: Supplementary file 1 — Additional file 1: Table S1. 27 Sample combinations for differentially expressed gene analysis. [file 12863_2024_1227_MOESM1_ESM.docx]

Table S1 27 Sample combinations for differentially expressed gene analysis

| HA853_0_I vs HA853_0_N | HA853_4_I vs HA853_4_N | HA853_8_I vs HA853_8_N |
| --- | --- | --- |
| HA89_0_I vs HA89_0_N | HA89_4_I vs HA89_4_N | HA89_8_I vs HA89_8_N |
| RK416_0_I vs RK416_0_N | RK416_4_I vs RK416_4_N | RK416_8_I vs RK416_8_N |
| HA853_4_I vs HA853_0_I | HA853_8_I vs HA853_0_I | HA853_8_I vs HA853_4_I |
| HA89_4_I vs HA89_0_I | HA89_8_I vs HA89_4_I | HA89_8_I vs HA89_0_I |
| RK416_4_I vs RK416_0_I | RK416_8_I vs RK416_4_I | RK416_8_I vs RK416_0_I |
| HA853_0_I vs HA89_0_I | HA853_0_I vs RK416_0_I | RK416_0_I vs HA89_0_I |
| HA853_4_I vs HA89_4_I | HA853_4_I vs RK416_4_I | RK416_4_I vs HA89_4_I |
| HA853_8_I vs HA89_8_I | HA853_8_I vs RK416_8_I | RK416_8_I vs HA89_8_I |

Each sample in this table was represented by Ils_time points_ inoculation treatments. HA853, HA89 and RK416 were name of three inbred lines (Ils). 0, 4 and 8 represent 0, 4, 8 days after inoculation, I and N represent pathogen inoculated and mock- (water) inoculated.
