# Supplementary material for: Pangenome characterization and analysis of the NAC gene family reveals genes for Sclerotinia sclerotiorum resistance in sunflower (Helianthus annuus)
Source: BMC Genom Data. 2024 May 1;25:39. doi: 10.1186/s12863-024-01227-9 (PMC11064331; doi:10.1186/s12863-024-01227-9)
Supplement: Supplementary file 2 — Additional file 2: Table S2. List of NAC genes of sunflower retrieved from the sunflower pan-genome database. [file 12863_2024_1227_MOESM2_ESM.docx]

Table S2 List of NAC genes of sunflower retrieved from the sunflower pan-genome database

| Name | Gene id | Type |
| --- | --- | --- |
| HaNAC1 | Ha1_00043854 | core |
| HaNAC2 | Ha1_00043855 | core |
| HaNAC3 | Ha1_00043887 | core |
| HaNAC4 | Ha1_00044272 | core |
| HaNAC5 | Ha1_00045074 | core |
| HaNAC6 | Ha1_00045124 | core |
| HaNAC7 | Ha1_00045163 | core |
| HaNAC8 | Ha2_00026421 | core |
| HaNAC9 | Ha2_00026737 | core |
| HaNAC10 | Ha2_00026738 | core |
| HaNAC11 | Ha2_00027457 | core |
| HaNAC12 | Ha2_00027535 | core |
| HaNAC13 | Ha2_00027639 | core |
| HaNAC14 | Ha2_00027886 | dispensable |
| HaNAC15 | Ha2_00028056 | core |
| HaNAC16 | Ha2_00028071 | core |
| HaNAC17 | Ha2_00028164 | dispensable |
| HaNAC18 | Ha2_00028186 | rare |
| HaNAC19 | Ha2_00028308 | core |
| HaNAC20 | Ha3_00035069 | dispensable |
| HaNAC21 | Ha3_00035258 | core |
| HaNAC22 | Ha3_00036100 | core |
| HaNAC23 | Ha3_00037104 | core |
| HaNAC24 | Ha3_00037297 | core |
| HaNAC25 | Ha4_00024436 | dispensable |
| HaNAC26 | Ha4_00025071 | dispensable |
| HaNAC27 | Ha4_00025271 | dispensable |
| HaNAC28 | Ha4_00025342 | core |
| HaNAC29 | Ha4_00025764 | core |
| HaNAC30 | Ha5_00006791 | core |
| HaNAC31 | Ha5_00006792 | rare |
| HaNAC32 | Ha5_00007352 | core |
| HaNAC33 | Ha5_00007366 | dispensable |
| HaNAC34 | Ha5_00007461 | core |
| HaNAC35 | Ha6_00047745 | core |
| HaNAC36 | Ha6_00048298 | core |
| HaNAC37 | Ha7_00045583 | core |
| HaNAC38 | Ha7_00045588 | core |
| HaNAC39 | Ha7_00045857 | dispensable |
| HaNAC40 | Ha7_00045863 | core |
| HaNAC41 | Ha7_00045942 | core |
| HaNAC42 | Ha7_00046178 | core |
| HaNAC43 | Ha7_00046827 | core |
| HaNAC44 | Ha7_00046828 | core |
| HaNAC45 | Ha7_00047113 | core |
| HaNAC46 | Ha8_00040929 | core |
| HaNAC47 | Ha8_00041007 | core |
| HaNAC48 | Ha8_00041472 | core |
| HaNAC49 | Ha8_00041473 | core |
| HaNAC50 | Ha8_00042181 | dispensable |
| HaNAC51 | Ha8_00042684 | core |
| HaNAC52 | Ha8_00042935 | core |
| HaNAC53 | Ha9_00011213 | dispensable |
| HaNAC54 | Ha9_00011347 | core |
| HaNAC55 | Ha9_00011532 | rare |
| HaNAC56 | Ha9_00012389 | core |
| HaNAC57 | Ha9_00012491 | core |
| HaNAC58 | Ha9_00012511 | rare |
| HaNAC59 | Ha9_00013140 | core |
| HaNAC60 | Ha9_00013179 | core |
| HaNAC61 | Ha9_00013691 | rare |
| HaNAC62 | Ha9_00014013 | core |
| HaNAC63 | Ha10_00000136 | core |
| HaNAC64 | Ha10_00000411 | core |
| HaNAC65 | Ha10_00001607 | core |
| HaNAC66 | Ha10_00002537 | core |
| HaNAC67 | Ha10_00003079 | core |
| HaNAC68 | Ha10_00003237 | core |
| HaNAC69 | Ha10_00004184 | core |
| HaNAC70 | Ha11_00028562 | core |
| HaNAC71 | Ha11_00029970 | core |
| HaNAC72 | Ha11_00030180 | core |
| HaNAC73 | Ha11_00030421 | core |
| HaNAC74 | Ha11_00030496 | dispensable |
| HaNAC75 | Ha11_00030573 | core |
| HaNAC76 | Ha12_00031518 | core |
| HaNAC77 | Ha12_00031626 | core |
| HaNAC78 | Ha12_00032103 | core |
| HaNAC79 | Ha12_00032106 | core |
| HaNAC80 | Ha12_00032132 | core |
| HaNAC81 | Ha12_00032142 | core |
| HaNAC82 | Ha12_00033111 | core |
| HaNAC83 | Ha12_00033273 | core |
| HaNAC84 | Ha12_00033617 | core |
| HaNAC85 | Ha12_00033708 | core |
| HaNAC86 | Ha13_00014332 | dispensable |
| HaNAC87 | Ha13_00014702 | core |
| HaNAC88 | Ha13_00014928 | core |
| HaNAC89 | Ha13_00015007 | core |
| HaNAC90 | Ha13_00015011 | core |
| HaNAC91 | Ha13_00015262 | core |
| HaNAC92 | Ha13_00015625 | dispensable |
| HaNAC93 | Ha13_00015845 | core |
| HaNAC94 | Ha13_00015847 | core |
| HaNAC95 | Ha13_00015849 | core |
| HaNAC96 | Ha13_00016409 | core |
| HaNAC97 | Ha13_00016537 | core |
| HaNAC98 | Ha13_00016764 | core |
| HaNAC99 | Ha13_00016785 | core |
| HaNAC100 | Ha14_00017639 | core |
| HaNAC101 | Ha14_00018609 | core |
| HaNAC102 | Ha14_00018968 | core |
| HaNAC103 | Ha14_00019719 | core |
| HaNAC104 | Ha14_00020126 | core |
| HaNAC105 | Ha14_00020127 | core |
| HaNAC106 | Ha14_00020430 | core |
| HaNAC107 | Ha15_00037878 | core |
| HaNAC108 | Ha15_00038230 | core |
| HaNAC109 | Ha15_00038844 | dispensable |
| HaNAC110 | Ha15_00038861 | core |
| HaNAC111 | Ha15_00039289 | core |
| HaNAC112 | Ha15_00039356 | core |
| HaNAC113 | Ha15_00039409 | core |
| HaNAC114 | Ha15_00039544 | core |
| HaNAC115 | Ha15_00039592 | core |
| HaNAC116 | Ha15_00040178 | dispensable |
| HaNAC117 | Ha15_00040242 | core |
| HaNAC118 | Ha15_00040407 | core |
| HaNAC119 | Ha15_00040655 | core |
| HaNAC120 | Ha15_00040662 | core |
| HaNAC121 | Ha16_00021072 | core |
| HaNAC122 | Ha16_00021532 | dispensable |
| HaNAC123 | Ha16_00021774 | dispensable |
| HaNAC124 | Ha16_00021775 | dispensable |
| HaNAC125 | Ha16_00022052 | dispensable |
| HaNAC126 | Ha16_00022085 | core |
| HaNAC127 | Ha16_00022215 | core |
| HaNAC128 | Ha16_00022465 | dispensable |
| HaNAC129 | Ha16_00022567 | core |
| HaNAC130 | Ha16_00023593 | core |
| HaNAC131 | Ha17_00008177 | core |
| HaNAC132 | Ha17_00008516 | core |
| HaNAC133 | Ha17_00008624 | core |
| HaNAC134 | Ha17_00008822 | core |
| HaNAC135 | Ha17_00008954 | core |
| HaNAC136 | Ha17_00010171 | core |
| HaNAC137 | Ha17_00010173 | core |
| HaNAC138 | Ha17_00010525 | core |
| HaNAC139 | A5Z0S7_CHRLV | core |
