# Supplementary material for: Pangenome characterization and analysis of the NAC gene family reveals genes for Sclerotinia sclerotiorum resistance in sunflower (Helianthus annuus)
Source: BMC Genom Data. 2024 May 1;25:39. doi: 10.1186/s12863-024-01227-9 (PMC11064331; doi:10.1186/s12863-024-01227-9)
Supplement: Supplementary file 4 — Additional file 4: Table S4. The number of SNPs and haplotypes in sunflower NAC gene family. [file 12863_2024_1227_MOESM4_ESM.docx]

Table S4 The number of SNPs and haplotypes in sunflower NAC gene family

| Name | SNP number |  | gcHap number |
| --- | --- | --- | --- |
| HaNAC1 | 21 |  | 174 |
| HaNAC2 | 2 |  | 9 |
| HaNAC3 | 33 |  | 206 |
| HaNAC4 | 36 |  | 171 |
| HaNAC5 | 38 |  | 246 |
| HaNAC6 | 32 |  | 213 |
| HaNAC7 | 21 |  | 203 |
| HaNAC8 | 37 |  | 187 |
| HaNAC9 | 11 |  | 109 |
| HaNAC10 | 17 |  | 128 |
| HaNAC11 | 8 |  | 78 |
| HaNAC12 | 38 |  | 215 |
| HaNAC13 | 40 |  | 245 |
| HaNAC14 | 12 |  | 110 |
| HaNAC15 | 32 |  | 228 |
| HaNAC16 | 49 |  | 269 |
| HaNAC17 | 17 |  | 151 |
| HaNAC18 | 11 |  | 80 |
| HaNAC19 | 0 |  | 0 |
| HaNAC20 | 0 |  | 0 |
| HaNAC21 | 26 |  | 183 |
| HaNAC22 | 0 |  | 0 |
| HaNAC23 | 42 |  | 242 |
| HaNAC24 | 38 |  | 204 |
| HaNAC25 | 0 |  | 0 |
| HaNAC26 | 0 |  | 0 |
| HaNAC27 | 0 |  | 0 |
| HaNAC28 | 28 |  | 175 |
| HaNAC29 | 27 |  | 176 |
| HaNAC30 | 40 |  | 213 |
| HaNAC31 | 10 |  | 77 |
| HaNAC32 | 20 |  | 153 |
| HaNAC33 | 12 |  | 102 |
| HaNAC34 | 74 |  | 275 |
| HaNAC35 | 55 |  | 242 |
| HaNAC36 | 0 |  | 0 |
| HaNAC37 | 44 |  | 239 |
| HaNAC38 | 32 |  | 231 |
| HaNAC39 | 9 |  | 71 |
| HaNAC40 | 30 |  | 232 |
| HaNAC41 | 0 |  | 0 |
| HaNAC42 | 0 |  | 0 |
| HaNAC43 | 19 |  | 213 |
| HaNAC44 | 43 |  | 252 |
| HaNAC45 | 0 |  | 0 |
| HaNAC46 | 0 |  | 0 |
| HaNAC47 | 22 |  | 180 |
| HaNAC48 | 42 |  | 238 |
| HaNAC49 | 43 |  | 276 |
| HaNAC50 | 9 |  | 115 |
| HaNAC51 | 26 |  | 189 |
| HaNAC52 | 28 |  | 184 |
| HaNAC53 | 0 |  | 0 |
| HaNAC54 | 0 |  | 0 |
| HaNAC55 | 0 |  | 0 |
| HaNAC56 | 1 |  | 3 |
| HaNAC57 | 19 |  | 211 |
| HaNAC58 | 0 |  | 0 |
| HaNAC59 | 51 |  | 263 |
| HaNAC60 | 48 |  | 255 |
| HaNAC61 | 0 |  | 0 |
| HaNAC62 | 6 |  | 47 |
| HaNAC63 | 72 |  | 299 |
| HaNAC64 | 31 |  | 194 |
| HaNAC65 | 21 |  | 149 |
| HaNAC66 | 0 |  | 0 |
| HaNAC67 | 23 |  | 191 |
| HaNAC68 | 11 |  | 136 |
| HaNAC69 | 61 |  | 310 |
| HaNAC70 | 36 |  | 172 |
| HaNAC71 | 42 |  | 232 |
| HaNAC72 | 15 |  | 136 |
| HaNAC73 | 0 |  | 0 |
| HaNAC74 | 0 |  | 0 |
| HaNAC75 | 24 |  | 189 |
| HaNAC76 | 58 |  | 270 |
| HaNAC77 | 26 |  | 185 |
| HaNAC78 | 18 |  | 137 |
| HaNAC79 | 6 |  | 62 |
| HaNAC80 | 34 |  | 200 |
| HaNAC81 | 18 |  | 143 |
| HaNAC82 | 0 |  | 0 |
| HaNAC83 | 40 |  | 219 |
| HaNAC84 | 16 |  | 153 |
| HaNAC85 | 37 |  | 254 |
| HaNAC86 | 16 |  | 123 |
| HaNAC87 | 57 |  | 219 |
| HaNAC88 | 25 |  | 180 |
| HaNAC89 | 35 |  | 213 |
| HaNAC90 | 0 |  | 0 |
| HaNAC91 | 53 |  | 252 |
| HaNAC92 | 23 |  | 147 |
| HaNAC93 | 53 |  | 166 |
| HaNAC94 | 32 |  | 212 |
| HaNAC95 | 28 |  | 201 |
| HaNAC96 | 57 |  | 223 |
| HaNAC97 | 43 |  | 241 |
| HaNAC98 | 8 |  | 77 |
| HaNAC99 | 16 |  | 143 |
| HaNAC100 | 24 |  | 158 |
| HaNAC101 | 40 |  | 212 |
| HaNAC102 | 46 |  | 220 |
| HaNAC103 | 40 |  | 227 |
| HaNAC104 | 21 |  | 174 |
| HaNAC105 | 52 |  | 238 |
| HaNAC106 | 16 |  | 134 |
| HaNAC107 | 17 |  | 176 |
| HaNAC108 | 29 |  | 200 |
| HaNAC109 | 10 |  | 127 |
| HaNAC110 | 0 |  | 0 |
| HaNAC111 | 42 |  | 210 |
| HaNAC112 | 45 |  | 262 |
| HaNAC113 | 0 |  | 0 |
| HaNAC114 | 0 |  | 0 |
| HaNAC115 | 33 |  | 209 |
| HaNAC116 | 0 |  | 0 |
| HaNAC117 | 3 |  | 14 |
| HaNAC118 | 24 |  | 189 |
| HaNAC119 | 58 |  | 262 |
| HaNAC120 | 0 |  | 0 |
| HaNAC121 | 35 |  | 221 |
| HaNAC122 | 0 |  | 0 |
| HaNAC123 | 17 |  | 212 |
| HaNAC124 | 10 |  | 158 |
| HaNAC125 | 0 |  | 0 |
| HaNAC126 | 1 |  | 3 |
| HaNAC127 | 25 |  | 209 |
| HaNAC128 | 7 |  | 68 |
| HaNAC129 | 0 |  | 0 |
| HaNAC130 | 77 |  | 242 |
| HaNAC131 | 25 |  | 107 |
| HaNAC132 | 9 |  | 106 |
| HaNAC133 | 66 |  | 256 |
| HaNAC134 | 69 |  | 213 |
| HaNAC135 | 45 |  | 279 |
| HaNAC136 | 0 |  | 0 |
| HaNAC137 | 12 |  | 126 |
| HaNAC138 | 15 |  | 130 |
| HaNAC139 | 0 |  | 0 |
