# Supplementary material for: Pangenome characterization and analysis of the NAC gene family reveals genes for Sclerotinia sclerotiorum resistance in sunflower (Helianthus annuus)
Source: BMC Genom Data. 2024 May 1;25:39. doi: 10.1186/s12863-024-01227-9 (PMC11064331; doi:10.1186/s12863-024-01227-9)
Supplement: Supplementary file 5 — Additional file 5: Table S5. 26. Differentially expressed HaNAC genes. [file 12863_2024_1227_MOESM5_ESM.docx]

Table S5 26 Differentially expressed *HaNAC* genes

| **Gene** | **baseMean** | **Log2FoldChange** | **pval** | **padj** | **Up/Down** | **Group** |
| --- | --- | --- | --- | --- | --- | --- |
| HaNAC75 | 787.2873856 | -4.861328907 | 7.83437E-18 | 1.09293E-15 | down | HA853_4_I-HA853_0_I |
| HaNAC79 | 505.0567064 | -1.293171985 | 9.66166E-07 | 1.93573E-05 | down | HA853_4_I-HA853_0_I |
| HaNAC80 | 497.9252475 | 2.873587353 | 1.33E-18 | 1.99E-16 | up | HA853_4_I-HA853_0_I |
| HaNAC89 | 41.53798159 | 1.450413657 | 2.21E-03 | 1.54E-02 | up | HA853_4_I-HA853_0_I |
| HaNAC93 | 19.82509835 | 5.587460075 | 1.23E-07 | 3.12E-06 | up | HA853_4_I-HA853_0_I |
| HaNAC108 | 1318.506652 | 1.673070084 | 3.87E-29 | 2.40E-26 | up | HA853_4_I-HA853_0_I |
| HaNAC34 | 335.2910789 | 1.082532089 | 1.27E-06 | 2.49E-05 | up | HA853_4_I-HA853_0_I |
| HaNAC54 | 47.06819856 | 1.354688272 | 7.04E-03 | 3.96E-02 | up | HA853_4_I-HA853_0_I |
| HaNAC68 | 55.65546057 | 2.48375037 | 4.71E-07 | 4.03E-06 | up | HA853_8_I-HA853_0_I |
| HaNAC75 | 773.5963571 | -5.823821573 | 6.67831E-33 | 4.08527E-31 | down | HA853_8_I-HA853_0_I |
| HaNAC80 | 546.0579093 | 3.04117309 | 1.02E-23 | 3.99E-22 | up | HA853_8_I-HA853_0_I |
| HaNAC89 | 70.83924324 | 2.527202138 | 6.01426E-09 | 6.5531E-08 | up | HA853_8_I-HA853_0_I |
| HaNAC93 | 8.595258224 | 4.340097476 | 8.10E-04 | 0.003887488 | up | HA853_8_I-HA853_0_I |
| HaNAC94 | 45.4492115 | 2.549960646 | 3.95E-07 | 3.40304E-06 | up | HA853_8_I-HA853_0_I |
| HaNAC108 | 1490.618335 | 1.937708633 | 3.67E-32 | 2.17E-30 | up | HA853_8_I-HA853_0_I |
| HaNAC119 | 220.1542756 | 1.028418522 | 0.000331625 | 0.001737706 | up | HA853_8_I-HA853_0_I |
| HaNAC28 | 110.9123967 | -1.441717539 | 2.27E-03 | 9.76E-03 | down | HA853_8_I-HA853_0_I |
| HaNAC34 | 365.6421729 | 1.299393861 | 4.45E-10 | 5.68E-09 | up | HA853_8_I-HA853_0_I |
| HaNAC40 | 101.9758077 | 3.07900641 | 5.5671E-05 | 0.00033865 | up | HA853_8_I-HA853_0_I |
| HaNAC51 | 13.95541044 | 3.947013325 | 6.33771E-05 | 0.000381267 | up | HA853_8_I-HA853_0_I |
| HaNAC54 | 44.93024141 | 1.26567415 | 0.015004285 | 0.048856656 | up | HA853_8_I-HA853_0_I |
| HaNAC60 | 1754.653625 | 2.343058242 | 1.08E-19 | 3.35E-18 | up | HA853_8_I-HA853_0_I |
| HaNAC94 | 57.66538043 | 2.194828429 | 3.65E-04 | 5.21E-03 | up | HA853_8_I-HA853_4_I |
| HaNAC40 | 141.5989989 | 1.962598409 | 1.05E-04 | 1.76E-03 | up | HA853_8_I-HA853_4_I |
| HaNAC60 | 2439.91573 | 1.392723469 | 1.01E-09 | 5.61E-08 | up | HA853_8_I-HA853_4_I |
| HaNAC75 | 648.5902562 | -3.852478982 | 2.27E-24 | 5.15E-22 | down | HA89_4_I-HA89_0_I |
| HaNAC80 | 259.4287039 | 3.344954303 | 4.24E-25 | 1.07E-22 | up | HA89_4_I-HA89_0_I |
| HaNAC83 | 312.7153328 | 1.706254904 | 2.61E-16 | 2.52E-14 | up | HA89_4_I-HA89_0_I |
| HaNAC89 | 25.89929393 | 1.837882458 | 0.000248461 | 0.002719635 | up | HA89_4_I-HA89_0_I |
| HaNAC107 | 10.21401822 | 2.548511678 | 8.73E-04 | 7.90E-03 | up | HA89_4_I-HA89_0_I |
| HaNAC108 | 934.8451992 | 1.474263933 | 6.91885E-07 | 1.60191E-05 | up | HA89_4_I-HA89_0_I |
| HaNAC39 | 7.47089109 | 3.621991324 | 0.006242724 | 0.039408334 | up | HA89_4_I-HA89_0_I |
| HaNAC60 | 1255.771843 | 1.861512136 | 4.80E-07 | 1.14707E-05 | up | HA89_4_I-HA89_0_I |
| HaNAC89 | 90.00553201 | 1.870677319 | 7.02342E-06 | 0.000134916 | up | HA89_8_I-HA89_4_I |
| HaNAC94 | 66.1566568 | 2.3157337 | 1.12E-06 | 2.51E-05 | up | HA89_8_I-HA89_4_I |
| HaNAC108 | 1945.254919 | 1.018886822 | 6.36E-11 | 2.69E-09 | up | HA89_8_I-HA89_4_I |
| HaNAC119 | 152.7077349 | 1.137949178 | 2.27E-03 | 2.18E-02 | up | HA89_8_I-HA89_4_I |
| HaNAC60 | 2898.130559 | 1.017427731 | 8.25E-12 | 3.97E-10 | up | HA89_8_I-HA89_4_I |
| HaNAC63 | 63.4108875 | 1.307075155 | 1.41E-03 | 6.70E-03 | up | HA89_8_I-HA89_0_I |
| HaNAC68 | 34.92737221 | 2.035801039 | 8.13E-06 | 6.57E-05 | up | HA89_8_I-HA89_0_I |
| HaNAC75 | 507.0125477 | -4.941702662 | 5.52E-29 | 4.33394E-27 | down | HA89_8_I-HA89_0_I |
| HaNAC77 | 142.7432562 | 1.145565091 | 6.42861E-07 | 6.36404E-06 | up | HA89_8_I-HA89_0_I |
| HaNAC80 | 332.128453 | 4.132159233 | 5.42191E-36 | 5.55584E-34 | up | HA89_8_I-HA89_0_I |
| HaNAC83 | 220.1464768 | 1.415274184 | 3.5424E-09 | 5.1418E-08 | up | HA89_8_I-HA89_0_I |
| HaNAC89 | 54.39960009 | 3.699345222 | 9.51492E-19 | 3.91554E-17 | up | HA89_8_I-HA89_0_I |
| HaNAC93 | 12.1024901 | 5.319619076 | 1.47886E-06 | 1.36775E-05 | up | HA89_8_I-HA89_0_I |
| HaNAC94 | 42.13602867 | 3.652962146 | 6.73553E-16 | 2.18488E-14 | up | HA89_8_I-HA89_0_I |
| HaNAC107 | 9.999886324 | 2.944956244 | 0.000287332 | 0.001650904 | up | HA89_8_I-HA89_0_I |
| HaNAC108 | 1173.131505 | 2.466525211 | 2.10394E-16 | 7.21824E-15 | up | HA89_8_I-HA89_0_I |
| HaNAC119 | 113.3347598 | 1.653321168 | 6.68177E-06 | 5.47021E-05 | up | HA89_8_I-HA89_0_I |
| HaNAC29 | 56.27534025 | 1.446932971 | 2.90403E-05 | 0.000211454 | up | HA89_8_I-HA89_0_I |
| HaNAC34 | 249.9493205 | 1.429476574 | 2.63E-04 | 1.53E-03 | up | HA89_8_I-HA89_0_I |
| HaNAC39 | 6.596447597 | 3.819270419 | 0.0066978 | 0.025500751 | up | HA89_8_I-HA89_0_I |
| HaNAC40 | 79.68825227 | 2.458251964 | 1.32E-05 | 1.02E-04 | up | HA89_8_I-HA89_0_I |
| HaNAC54 | 39.72199871 | 2.681313317 | 5.80608E-07 | 5.78477E-06 | up | HA89_8_I-HA89_0_I |
| HaNAC60 | 1636.818855 | 2.851788 | 8.33463E-15 | 2.41501E-13 | up | HA89_8_I-HA89_0_I |
| HaNAC68 | 200.7342684 | 2.337659224 | 3.06E-06 | 4.17E-05 | up | RK146_8_I-RK146_4_I |
| HaNAC75 | 176.5752175 | -3.481166084 | 3.82021E-16 | 2.99119E-14 | down | RK146_8_I-RK146_4_I |
| HaNAC79 | 586.9693125 | 1.289180005 | 4.46E-09 | 1.114E-07 | up | RK146_8_I-RK146_4_I |
| HaNAC80 | 680.4424397 | 1.085937701 | 0.000765263 | 0.005798132 | up | RK146_8_I-RK146_4_I |
| HaNAC89 | 113.5160184 | 2.000849203 | 4.32E-11 | 1.52E-09 | up | RK146_8_I-RK146_4_I |
| HaNAC94 | 107.8296122 | 1.557229366 | 8.61675E-14 | 4.67256E-12 | up | RK146_8_I-RK146_4_I |
| HaNAC107 | 15.44954873 | 2.887421253 | 1.94E-06 | 2.78E-05 | up | RK146_8_I-RK146_4_I |
| HaNAC33 | 56.77170469 | 1.428459091 | 0.000434054 | 0.0035637 | up | RK146_8_I-RK146_4_I |
| HaNAC54 | 46.93377964 | 1.518856692 | 2.25917E-06 | 3.18642E-05 | up | RK146_8_I-RK146_4_I |
| HaNAC60 | 2146.656495 | 1.17828906 | 1.19287E-08 | 2.76839E-07 | up | RK146_8_I-RK146_4_I |
| HaNAC68 | 186.2875045 | 2.667552834 | 1.33771E-05 | 0.000784009 | up | RK146_8_I-RK146_0_I |
| HaNAC60 | 2100.424821 | 1.091836624 | 0.001451506 | 0.019304156 | up | RK146_8_I-RK146_0_I |
| HaNAC80 | 93.04520876 | 1.002695878 | 0.005687846 | 0.035246436 | up | HA853_0_I-HA89_0_I |
| HaNAC5 | 3.331843597 | -4.979360982 | 0.00203947 | 0.01552067 | down | HA853_0_I-RK146_0_I |
| HaNAC28 | 87.65629074 | 1.809204029 | 5.98246E-05 | 0.000831903 | up | HA853_0_I-RK146_0_I |
| HaNAC119 | 150.9663566 | 1.372631613 | 5.51E-04 | 0.00511185 | up | RK146_0_I-HA89_0_I |
| HaNAC22 | 15.02670336 | 1.993047541 | 3.05706E-05 | 0.000409438 | up | RK146_0_I-HA89_0_I |
| HaNAC28 | 94.94886772 | -1.901653059 | 1.13177E-06 | 2.14347E-05 | down | RK146_0_I-HA89_0_I |
| HaNAC31 | 8.417619937 | 2.792608227 | 0.001395961 | 0.011059249 | up | RK146_0_I-HA89_0_I |
| HaNAC51 | 5.142637656 | 4.996300869 | 0.000391358 | 0.003856208 | up | RK146_0_I-HA89_0_I |
| HaNAC93 | 31.66419041 | 1.911493829 | 0.004882467 | 0.041097102 | up | HA853_4_I-HA89_4_I |
| HaNAC75 | 238.8818221 | -2.834778159 | 6.57811E-06 | 9.52533E-05 | down | HA853_4_I-RK146_4_I |
| HaNAC80 | 669.3061733 | 1.292242522 | 0.002064497 | 0.013399869 | up | HA853_4_I-RK146_4_I |
| HaNAC93 | 20.28765996 | 3.474429539 | 1.93354E-08 | 5.04645E-07 | up | HA853_4_I-RK146_4_I |
| HaNAC94 | 45.16080141 | -1.399542651 | 0.000877959 | 0.0065967 | down | HA853_4_I-RK146_4_I |
| HaNAC5 | 8.283404744 | -6.03946764 | 0.000223207 | 0.002063233 | down | HA853_4_I-RK146_4_I |
| HaNAC22 | 34.19696514 | -1.571244285 | 0.001403985 | 0.009780419 | down | HA853_4_I-RK146_4_I |
| HaNAC28 | 67.19175535 | 2.024497458 | 1.03508E-07 | 2.31409E-06 | up | HA853_4_I-RK146_4_I |
| HaNAC40 | 35.07405473 | 1.406301123 | 0.010275453 | 0.048186027 | up | HA853_4_I-RK146_4_I |
| HaNAC54 | 43.18884211 | 1.663820928 | 1.28349E-05 | 0.000172568 | up | HA853_4_I-RK146_4_I |
| HaNAC75 | 250.588124 | 2.168968449 | 7.44738E-06 | 0.000116052 | up | RK146_4_I-HA89_4_I |
| HaNAC107 | 11.24188302 | -2.758543855 | 0.000664522 | 0.005789258 | down | RK146_4_I-HA89_4_I |
| HaNAC28 | 80.07404191 | -2.367235424 | 1.97283E-11 | 9.16329E-10 | down | RK146_4_I-HA89_4_I |
| HaNAC39 | 10.7065486 | -2.218746731 | 0.001984834 | 0.014536743 | down | RK146_4_I-HA89_4_I |
| HaNAC40 | 56.63345903 | -2.438728262 | 1.97202E-06 | 3.53115E-05 | down | RK146_4_I-HA89_4_I |
| HaNAC54 | 35.63859034 | -1.147042058 | 0.006130626 | 0.036618239 | down | RK146_4_I-HA89_4_I |
| HaNAC60 | 1850.903345 | -1.022516849 | 2.1609E-06 | 3.80853E-05 | down | RK146_4_I-HA89_4_I |
| HaNAC51 | 12.16120042 | 4.438021681 | 0.003300929 | 0.036270918 | up | HA853_8_I-HA89_8_I |
| HaNAC5 | 11.43766655 | -6.583230752 | 0.000101311 | 0.001322007 | down | HA853_8_I-RK146_8_I |
| HaNAC40 | 110.0215479 | 1.961459535 | 0.001648225 | 0.013521033 | up | HA853_8_I-RK146_8_I |
| HaNAC50 | 5.76292304 | -5.618322461 | 0.001461339 | 0.012258668 | down | HA853_8_I-RK146_8_I |
| HaNAC68 | 196.2584326 | 1.75418236 | 0.003691686 | 0.025721268 | up | RK146_8_I-HA89_8_I |
| HaNAC93 | 15.28728664 | -2.902905312 | 1.05492E-05 | 0.000198969 | down | RK146_8_I-HA89_8_I |
| HaNAC28 | 44.16726653 | -1.973083933 | 0.000474987 | 0.004989706 | down | RK146_8_I-HA89_8_I |
| HaNAC40 | 92.2341738 | -2.060434654 | 0.000587458 | 0.005926105 | down | RK146_8_I-HA89_8_I |
| HaNAC51 | 14.80729002 | 4.157061348 | 0.000612589 | 0.006116878 | up | RK146_8_I-HA89_8_I |
